# AN EMPIRICAL TYPOLOGY OF CULTURE CHANGE IMPLEMENTATION IN NURSING HOMES

**DOI:** 10.1093/geroni/igz038.2949

**Published:** 2019-11-08

**Authors:** Yinfei Duan, Christine Mueller

**Affiliations:** 1 School of Nursing, University of Minnesota, Minneapolis, Minnesota, United States; 2 University of Minnesota, Minneapolis, Minnesota, United States

## Abstract

The implementation of culture change (CC) practices has been increasing in U.S. nursing homes (NHs) with varying success. No study has examined the typology of CC implementation across NHs using empirical data. This study aimed to identify an empirical typology of CC implementation and to examine NH organizational characteristics associated with types of CC implementation. An online CC survey using a validated instrument was sent to administrators of 363 NHs in Minnesota and 78 administrators completed the survey. No significant difference in NH characteristics was found between participants and non-participants. Using a hierarchical cluster analysis based on scores of five CC domains, this study identified three distinctive clusters. Overall, NHs most frequently engaged in resident-centered care and environment transformation and least frequently implemented family/community engagement practices. While NHs in cluster 1 (20.8%) performed best in all CC domains, they were distinguished from others by more frequency of family/community engagement, staff empowerment and staff leadership practices. NHs in cluster 3 (41.7%) were distinguished by overall lower engagement of CC practices in all domains. While NHs in cluster 2 (37.5%) and cluster 1 were comparable in environment transformation and resident-centered care, NHs in cluster 2 and cluster 3 were similar in their low family/community engagement. NHs in cluster 3 had significantly lower registered nurse retention, and lower mental health and social service staffing compared to NHs in cluster 2. This typology provides insight into the variations in implementing CC practices across NHs and pragmatic implications for promoting deep and extensive CC.

